# Cognitive Structure of College Students and Teaching Strategies of Ideological and Political Education Under Educational Psychology

**DOI:** 10.3389/fpsyg.2022.892110

**Published:** 2022-06-15

**Authors:** Jing Zhao

**Affiliations:** School of Marxism, Northwestern Polytechnical University, Xi’an, China

**Keywords:** educational psychology, higher education, ideological and political, cognitive structure, educational strategy

## Abstract

The ideological and political education (IPE) situation is constantly developing and changing. In modern globalization, the ideological education of college students has received great attention. The purpose is to strengthen morality and cultivate people as the basic point of a college education. The principles of educational psychology are adopted to integrate IPE into the whole process of college teaching and help students develop healthily for a long time. First, IPE psychology’s concept and subject attribute under educational psychology are expounded. Next, the concept and development of cognitive structure theory are introduced. Moreover, educational constructivist measures are analyzed. Furthermore, the cognitive structure of college students’ self-cognition and IPE is interpreted and analyzed using cognitive psychology. Then, a questionnaire is designed to study the influencing factors of political education strategies in colleges. Finally, the questionnaire is collected to summarize the influencing factors and put forward optimization strategies. The results show that using the principles of educational psychology and cognitive psychology to investigate can accurately understand modern college students’ self-cognitive structure and ideological and political cognitive structure. The opportunity factor greatly impacts the IPE strategy of college students. More than 97% think that it has an impact, of which more than 51% think that it has a great impact and more than 21% think that it is the decisive factor. Challenge factors greatly impact college students’ IPE strategies. More than 97% think they have an impact, of which more than 55% think they have a great impact, and more than 24% think they are decisive factors. It shows that educational psychology is conducive to the progress of political education in colleges and the improvement of college students’ ideological and political levels. This exploration provides a new direction for educational psychology research in ideological and political work.

## Introduction

Xi Jinping pointed out: “to run a socialist college with Chinese characteristics well, it is essential to adhere to building morality and cultivating people, integrate the cultivation and practice of socialist core values into the whole process of teaching and educating people, strengthen ideological guidance and firmly grasp the leadership of ideological work in colleges.” Ideological and political education (IPE) in colleges plays a crucial role in the fundamental task of “Building Morality and cultivating people.” Colleges should put IPE in a more prominent position and adhere to the guiding position of Marxism. Therefore, only by strengthening the value recognition of college teachers and students for IPE, can the effectiveness of IPE in colleges be enhanced. In recent years, with the acceleration of economic globalization and the in-depth development of the trend of world multi-polarization, China’s economic interests also show a diversified development trend. Meanwhile, the impact of Western values has diversified the values of college teachers and students, which has brought severe challenges to the value identity of IPE in colleges. Besides, there are some problems in the IPE in colleges. The leaders of some colleges do not pay much attention to the status of IPE. The development of educators’ work is not in line with the reality of students and focuses on single theory teaching. The educational method is too simple, so that the IPE can not play a better role in value guidance and moral norms among college students. It makes some people doubt the role of IPE itself, and shakes the position of IPE in colleges. The IPE’ essence is a kind of value identity, that is, the value identity of the educated for the content of IPE. It includes the identity of political outlook, moral and legal outlook, outlook on life and values. Given this, it is vital to research the value identification of IPE in colleges. Thus, what is the structure of IPE value identity in colleges? What is its psychological mechanism? How to scientifically adopt IPE strategies to improve IPE effectiveness in colleges according to the characteristics of the structure and psychological mechanism of IPE value identification in colleges is the significance of this exploration. Educational psychology is a social psychology that studies people’s learning and the impact of educational measures in the educational environment. It studies the basic psychological laws of learning and teaching in this environment. Educational psychology has such characteristics. Based on the pedagogical system, it studies how to cultivate people with all-round development (Matthews and Lopez, 2019). People’s psychological structure and cognitive level must be studied first. Its system should be established according to the law of the research results, and then the psychological phenomena of family, school, and social education must be studied. Educational psychology is not independent of ordinary psychology. The theoretical knowledge and tools of general psychology can also be used in educational psychology. Educational psychology needs to pay attention to the analysis of students’ learning process and the study of students’ learning skills in class and after class (Agarwal, 2019). The primary task of educational psychology is to establish a reasonable theoretical system to improve teaching means and improve education levels to promote students to achieve better personal development (Jaffar et al., 2019).

In the new media era, Gao (2021) pointed out that there are some opportunities for the development of IPE and proposed a new teaching method from teaching concepts, teaching mechanisms, teaching resources, and teaching methods (Gao, 2021). Wang (2021) proposed a way to improve college students’ cognitive level by IPE, in which he integrated cultural confidence and the excellent traditional cultural spirit into IPE and exemplified the importance of cultural confidence for IPE, expanding the teaching content of IPE (Wang, 2021). Zhang et al. (2020) proposed a teaching method of IPE and moral education under virtual reality technology (VR), which helps students establish a good outlook on the world, life, and values (Zhang et al., 2020). Zhou and Wang (2020) explored the significance of learning habits, influencing factors, and educational practice of IPE, and discussed three learning behaviors: selecting learning materials, figuring out learning strategies (rereading and self-test), and updating strategies (how strategies are formed and changed) (Zhou and Wang, 2020). Lv et al. (2020) suggested a method based on deep learning (DL) to simulate and analyze college students’ IPE and give some suggestions for improving the teaching quality through analyzing human behavior and cognitive structure using advanced machine learning (ML) (Lv et al., 2020).

In short, the research on college students’ IPE focuses on: (a) updating teaching methods. (b) using more advanced behavior analysis tools. (c) using modern teaching means. The innovation is that a cognitive method of IPE is proposed based on educational psychology and the influencing factors of IPE. With the progress of reform and opening up, various ideas and thoughts spring up. Educational psychology is applied to the research of college students’ cognitive structures and the improvement strategies of IPE. First, educational psychology is introduced, and the psychology of IPE is discussed. Then, the cognitive structure of college students’ IPE is analyzed, and a questionnaire is designed based on the principle of educational psychology. The effects of IPE on college students’ cognition are analyzed. Finally, a questionnaire survey is conducted to analyze the influencing factors of IPE, and some teaching strategies are put forward. It is proved that the teaching method based on educational psychology can accurately analyze college students’ cognitive structure and the influencing factors of IPE. This study provides a new method for improving the teaching quality of IPE.

## College Students’ Cognitive Structure and the Relevant Teaching Strategies of Ideological and Political Education

### Research on Ideological and Political Education

#### Educational Psychology

Psychology reveals people’s psychological processes such as feelings, memories, thinking, emotions, needs, interests, motivations, and the general characteristics and laws of personality development. Educational psychology is the product of combining educational activities and psychologies. It is a science to explore the psychological laws in teaching and learning (Maestre, 2021). The research subjects of educational psychology are the psychological phenomena of students and teachers in education. It is not a subject that simply applies the general principles and methods of psychology to education (Priantini, 2021).

#### Psychological Elements in Ideological and Political Education

The psychology in IPE is the process of learning the basic political theories such as Marxism, Leninism, and Mao Zedong Thought, and correcting the ideological and moral character of educational subjects. Since the 1980s, the psychology in IPE has achieved systematic development and developed into an independent discipline. It comprehensively draws lessons from history, logic, pedagogy, psychology, sociology, and other disciplines, and becomes a discipline about Marxist theory. Its fundamental point is to explore people’s psychological changes (Tang and Shi, 2021). The differences between moral education and IPE are shown in Table 1.

**TABLE 1:** Difference between moral education and IPE.

	Moral education	IPE
Concept	Studying the formation and development process of people’s morality and the law of IPE	Revealing the mechanisms, laws, and nature of psychological phenomena in moral education.
Research subjects	Human psychology	Moral characters
Elements	Psychological mechanism, psychological laws, psychological phenomena, the psychological laws of the formation and development of people’s political ideas, and the performance, acceptance, and operation mechanism of psychological phenomena	Ideology, politics, legal system, and moral education

The psychology in IPE is a science that analyzes the formation and development of people’s ideology and morality and gives the corresponding strategies (Sun and Wang, 2020). It is a subject that reveals the psychological mechanism and development laws. Educational psychology is widely used in all aspects of social production and life. Since psychology and IPE study people’s thoughts and consciousness, the research on the psychology in IPE also needs to discuss people’s thoughts and consciousness (Zhang, 2021).

#### Discipline Attributes

Ideological and political education is an interdisciplinary subject of pedagogy and psychology. It belongs to the system of pedagogy and is a part of social ideology. It takes the Marxist Leninist Philosophy as the basis and the great rejuvenation of the Chinese nation as the Centennial goal. It is used for IPE and is ready to be tested by the practice of socialist educational construction. It serves for implementing the policy of socialism with Chinese characteristics (Ail, 2021).

### Cognitive Structures

#### The Concept of Cognition

**FIGURE 1:**
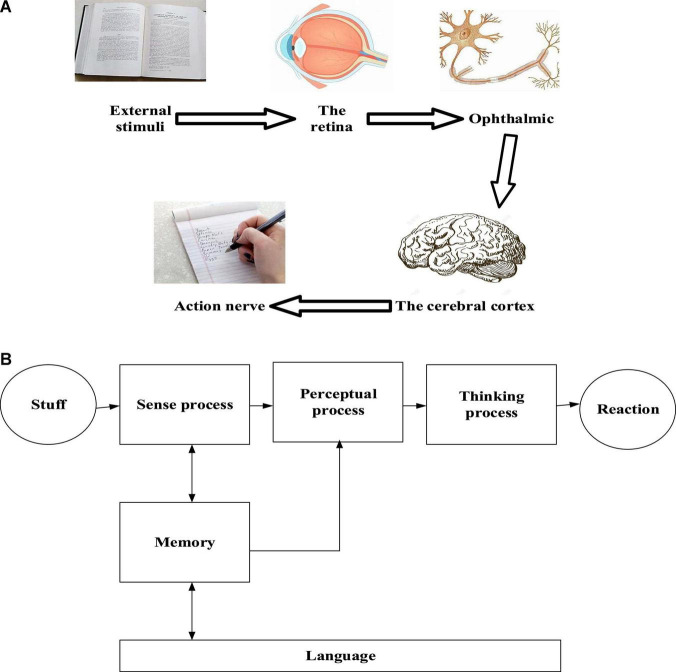
Cognitive process of human beings **(A)** physiological process **(B)** thinking process.

Cognition is how human beings accept or use knowledge or extract and process information. It is a basic thinking process. In detail, it is a process in which people convert the received stimulus into their psychological activities by brain processing to respond to the outside world after receiving external information. Feelings and perceptions are its foundation. Feeling is the understanding of the attributes and characteristics of things, while perception is the understanding of things as a whole and the relations of them. Therefore, it can be said that perceptions are based on feelings (Yang et al., 2020). Memory is perceptual experience and an essential basis for advanced cognitive processes such as thinking and language learning. When the external stimulus stops, the feeling or perception it brings will not necessarily disappear, but will be saved as individual experience (Kolobashkina and Alyushin, 2020). Thinking is complex, and it is produced by analyzing, synthesizing, comparing, abstracting, and summarizing the input information from the outside world. An analysis is the understanding of the attributes of things. Language is a symbol system formed by humans through highly structured sound combinations or writing symbols and gestures. It is also using the symbol system to exchange ideas (Shin, 2019). Figure 1 shows the cognitive process of human beings.

In Figure 1, cognition refers to the process of acquiring or applying knowledge, or the process of information processing, which is the most basic psychological process of people. In short, the cognitive process is the process of information addition, that is, the process in which people internalize the received external information into their own psychological activities after processing by the nervous system, and then dominate people’s external behavior. Cognitive activities include feeling, perception, memory, thinking and language. Modern cognitive psychology attaches great importance to and emphasizes the structural significance of cognition. The cognitive process is the process of processing new information and extracting and using stored information with the subject’s original knowledge structure to accept new knowledge.

The cognitive structure is the psychological expression of the concept, content, and structure. It is a psychological state when the external stimulus is received, and then correct cognition is formed. It falls into knowledge and experience (Boadu and Donnelly, 2019).

#### Cognitive Psychology

Cognitive psychology is a psychological trend and research direction in the West in the mid-1950s. It is a psychological branch targeted at how people obtain, store, transform, use and communicate information. Its main research subject is the cognitive process in human psychological phenomena. It is an essential branch of cognitive science (including computer science, communication science, linguistics, logic, and Anthropology) (Vada et al., 2020). Cognition is originally a common word in psychology. It is defined as a cognitive or knowledge process in a psychological dictionary, namely a rational and cognitive process relative to emotion, motivation, and will. It includes perception, representation, memory, and thinking, with thinking as its core (Bremer et al., 2020).

Cognitive psychology can be defined in broad and narrow senses. In a broad sense, it includes structuralism psychology, psychology, and information-processing psychology. In a narrow sense, it refers to information processing psychology. They emphasize the advanced process of research consciousness and understanding. Information processing psychology is the mainstream of American cognitive psychology, and it compares people with computers. The computer receives the input information from the surrounding environment and then processes, stores, and outputs it (Loth and Evans, 2019).

**FIGURE 2:**
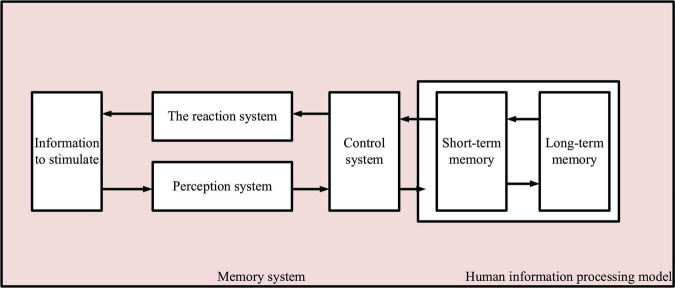
Information processing model.

Cognitive psychology believes that the human system is the same as the computer, and human acquisition of knowledge is also the process of information input, transformation, and storage. The specific cognitive behaviors at different stages are different. Information is temporarily stored in the original sensory form in the sensory system. It passes through the control system and enters short-term and long-term memory areas. The information stored in the long-term memory is extracted into the short-term memory, and the task is completed through problem-solving and decision-making (Till and Sproesser, 2020). In addition to the transformation from sensory storage to long-term memory, human information processing also displays the influence of the latter processing on the former processing. The former is called bottom-up, and the latter is top-down. Information processing is carried out in chronological order and in both directions (Jiang and Han, 2020), as shown in Figure 2.

Scientists believe that memory can be divided into short-term, medium-term, and long-term. The essence of short-term memory is the repetition of the brain’s immediate physiological and biochemical reactions, while medium-term and long-term memory is the structural changes and fixed connections in brain cells. Short-term memory is the largest and most unstable memory.

#### Elements of Cognitive Structures

**FIGURE 3:**
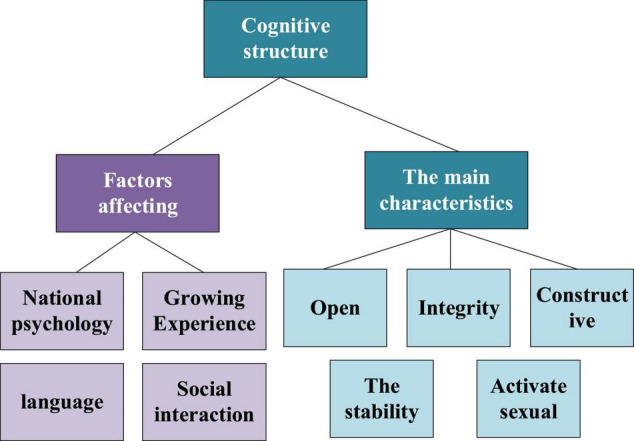
Overview of cognitive structure.

Table 2 shows the elements of the cognitive structure, and Figure 3 shows its overview.

**TABLE 2:** Elements of cognitive structures.

Name	Meanings	Sources	Effects
Knowledge	Practical experience and the laws of natural science and human science	Objective things, nature, and human society	It reflects people’s understandings of the content of the objective world
Structure	Knowledge structure	Knowledge	It provides background knowledge for the subject and views it as a reference for selecting, filtering, processing, and storing information, restricting the subject’s cognitive ways.
Cognitive styles	The cognitive way of the subjects in information processing	Self	It shows the differences between cognitive subjects and the influence of education.
Cognitive strategy	A collection of procedures, rules, methods, and techniques for indicating and detecting cognitive processes	Self	Cognition tools
Metacognition	Cognition about cognition	Self	Tools for understanding “cognition.”
Thinking modes	The way the human brain processes the information obtained	Self and human society	It shows how thinking activities are carried out, how human beings understand things and explore the angle, way, and method humans deal with things.
Irrational elements	The irrational elements are also needed, such as needs, emotions and will	Self	As a spiritual force, it penetrates into individual cognitive and practical activities

A model is an abstract representation of a system or a process. People’s information processing process model generally expresses people’s cognitive process. People’s response to external information usually needs to go through links like feeling, perception, memory, decision-making, response selection, and motor response. These links constitute a complete information processing system. Many researchers have proposed various models to express the human information processing process, and the more famous is the Broadbent model.

Value identity structure refers to the organic relationship between the main elements and their elements in the process of social members receiving value education and realizing value identity. It mainly includes four aspects: cognitive identity, emotional identity, internalized identity and behavioral identity. Cognitive identity is a process in which social members learn, accept, recognize, understand and comprehend some common values. It is a superficial knowledge understanding. In the process of IPE, it can be called theoretical indoctrination. Before making social members form core value identity, there should be a general stage of knowledge cognition, that is, the stage of feeling and perception mentioned in psychology. It has the characteristics of superficiality, transience and one-sidedness, which needs to be deepened in the process of continuous cognition. It is the starting point of value identity and the basis of emotional identity, internalization identity, and behavior identity. Emotional identity is social members’ emotional experience and subjective attitude based on cognitive values. From the perspective of psychology, people’s cognitive process and emotional processes are a process of mutual penetration and connection. In the cognitive stage, there will always be emotional interweaving. The formation of emotional identity is based on the knowledge of something. Generally, the identity subject can produce identity consciousness to the object that has a positive emotional experience. After the formation of emotional identity, it will greatly influence people’s cognition and become an important factor in cognitive activities. Internalized identity is that the value subject can actively and consciously accept and recognize some values and value objectives, and make them become their own value orientation. Internalized value concept is a core value concept based on specific value but beyond specific value.

### Construction of College Students’ Cognitive Structure

#### Self-Cognition

Self-cognition is the observation and understanding of the cognitive subject. It includes self-observation and self-evaluation (Gorbunova, 2020). When the cognitive subject has correct self-cognition, it indicates that he has a mature personality. Correct self-cognition plays the most important role in developing self-consciousness and college students’ psychological quality. Because college students are adolescents, they have strong self-esteem and are incredibly eager for success. However, they are prone to arrogance when they make an achievement. On the contrary, some college students feel largely invisible and have no sense of self-worth. Under this condition, their confidence and self-esteem will be lost when they feel frustrated, and they begin to doubt the world and life (Proctor et al., 2020).

It is common for college students to have cognitive disorders when improving their psychological states. In modern times, the communist belief is easy to be ruined by some bad opinions, behaviors, and activities. Therefore, it is particularly important to reconstruct college students’ self-cognition and help them have correct outlooks on values and life (Wang, 2020).

#### Constructivism Theory

The teaching objective of constructivism is to cultivate lifelong learners. Then, the learners can self-control the learning process and have the ability of self-analysis, self-evaluation, and self-reflection. The constructivist learning theory has a new view on knowledge and revolutionizes the traditional teaching mode. It transfers the teacher-centered teaching mode to the student-centered, which completely changes the traditional opinions on knowledge, learning, teaching, students, and teachers. Constructivism believes that the world exists objectively, but the understanding and meanings of the world are different among different people (Franco and Estevez, 2021). Everyone’s experience world is created with his own mind. Due to the difference in each person’s experience, each individual’s understanding of the external world is also very different (Kabir et al., 2021). Constructivists emphasize students’ active learning, but it also attaches importance to teachers’ help and guidance in the learning process. As for teachers, constructivists assign some new responsibilities for teachers (Pande and Bharathi, 2020), as shown in Table 3.

**TABLE 3:** Constructivists’ views on education.

View of knowledge	Learning view	Students view	Teaching view
A. Knowledge is not a purely objective reflection of reality, but an explanation, hypothesis or hypothesis of the objective world. In specific problem solving, original knowledge needs to be reprocessed and recreated according to the situation of specific problems.	A. Students need to adopt a new learning style, adopt a new cognitive processing strategy, and form a psychological model that they are the constructors of knowledge and understanding.	A. Since the meaning of things does not exist completely independently of us, but comes from our construction, and each person understands certain aspects of things in his own way, teaching should promote cooperation between students and enable students to see the basis of views that are different from his own.	A. The role of teachers should be changed from the traditional authority role of transferring knowledge to students to the mentor of students’ learning and become the senior collaborator of students’ learning.
B. Knowledge cannot exist as an entity outside the individual. Real understanding can only be constructed by learners themselves based on their own experience background and depends on the learning process in a specific context.	B. In constructivism teaching, teachers should always pay attention to keep learning tasks in the “zone of recent development” of students, and provide certain “supports” and guidance.	B. Teaching should make understanding richer and more comprehensive through the cooperation of learners.	B. Teachers are helpers and promoters of meaning construction, rather than providers and indoctrinators of knowledge. Students are the main body of learning information processing and the initiative of meaning construction, rather than the passive receiver of knowledge and the object of inculcation.
C. Textbook knowledge is only a reliable explanation or hypothesis about a phenomenon, rather than an “absolute reference” to explain the real world.	C. Students should realize the importance of becoming a self-control learner and try to learn some self-control skills and habits.	C. Teaching is not the transmission of knowledge, but the processing and conversion of knowledge.	C. Teachers should organize collaborative learning (discussion and communication) whenever possible, and guide the process of collaborative learning in a direction conducive to meaning construction.
D. Students’ reception of knowledge can only be constructed by themselves. They should analyze and judge the rationality of knowledge against the background of their own experience. In the learning process, students not only understand new knowledge, but also analyze, examine and criticize it.		D. In the teaching process, the learning content should be authentic and should not be oversimplified to make it far away from the realistic problem situation.	

**FIGURE 4:**
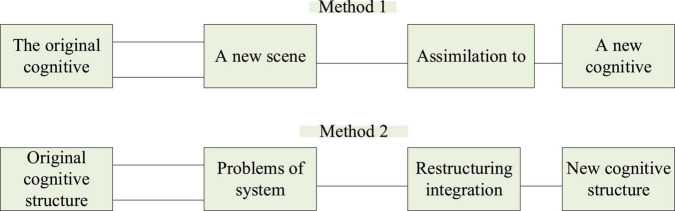
Cognitive construction process.

Figure 4 shows the cognitive construction process.

### Analysis of Teaching Strategies

#### Analysis of Opportunities, Threats, Strengths, and Weaknesses (SWOT)

SWOT is an analysis method under the internal and external competitive environment and competitive conditions. It lists and arranges the internal strengths, weaknesses, external opportunities, and threats and analyzes the various factors to perform decision-making (Jiang, 2021). Table 4 shows the SWOT analysis.

**TABLE 4:** The SWOT analysis.

	Strengths a. b. c.	Weaknesses a. b. c.
Opportunities a. b. c.	Opportunities- Strengths a. b. c.	Opportunities- Weaknesses a. b. c.
Threats a. b. c.	Threats- Strengths a. b. c.	Threats- Weaknesses a. b. c.

Strengths in the above table are internal advantages, weaknesses are internal disadvantages, opportunities are external advantages, and threads are external disadvantages.

#### Investigating the Influencing Factors

According to SWOT, the teaching strategies of college students’ IPE are analyzed, and the influencing factors of IPE are discussed by a questionnaire survey. Table 5 shows the evaluation indexes.

**TABLE 5:** Evaluation indexes.

Categories	Number	Indexes
Advantage	a	Technical equipment and facilities
	b	Excellent traditions and qualities
	c	Campus cultural activities
Disadvantage	a	Educational and teaching environments
	b	Controllability of the network environment
	c	Learning atmospheres
	d	The system of IPE
Opportunities	a	Teaching materials of IPE
	b	Reform of IPE
	c	Perfection of the socialist theory
	d	Attaching importance to IPE
Challenge	a	Employment environments
	b	Multiculturalism
	c	Market economy
	d	Family spiritual environments

### Questionnaire Design and Distribution

The first questionnaire of this exploration contains 38 questions. The scoring method adopts Likert’s five-point response scale. 1 means very inconformity, 2 means inconformity, 3 means uncertainty, 4 means conformity and 5 means very conformity. The second questionnaire consists of 14 questions, forming a semi-structured interview outline. The determination of each test question of each dimension follows the following principles. (1) Based on the empirical research items of others in this dimension, it is established as cognitive identity, emotional identity, internalized identity and behavioral identity. (2) The way and content of questioning conform to the habits of Chinese culture and the background characteristics of college students’ psychological activities. (3) If this dimension has no empirical research basis that others can learn from, it is essential to seek theoretical support, and then determine the number and content of measurement topics according to the theoretical basis.

The gender, age, educational background, and occupation of the respondents are investigated, and the influencing factors and the teaching environment of IPE are analyzed. The results are shown in Table 4. A 150 questionnaires are distributed by e-mail, and 139 are recovered, of which 127 are valid. A 50 copies are distributed by offline interviews, and 44 are recovered, including 41 valid questionnaires.

The reliability and validity of the questionnaire are analyzed. Reliability analysis is conducted to evaluate whether the respondents’ answers are reliable. For example, if the results of multiple measurements on the same item are very close, the value of this result will be considered reliable. If the results of each measurement are very different, it indicates that the reliability is low. Validity analysis is analyzing the validity and accuracy of the questionnaire. It is used to measure whether the items in the questionnaire are reasonable. Validity can be divided into content validity, structure validity, and criterion validity. Content validity presents the validity of the questionnaire in words. For example, the authority and effectiveness of the questionnaire are illustrated by references or authoritative sources (Ansari, 2021). The commonly used reliability coefficients include Cronbach’s alpha coefficients, half coefficients, and Kaiser Meyer Olkin (KMO), which can be analyzed by spssau.

Cronbach’s alpha coefficients are shown in Eq. 1, KMO coefficients are shown in Eq. 2, partial correlation coefficient *r*_*ij*(*k*)_ between *i*,*j* for variable *k* after control is shown in Eq. 3, and the test statistic of partial correlation analysis is shown in Eq. 4.


(1) $$\alpha =\frac{K}{K-1}\,\left(1-\frac{\sum S_{i}^{2}}{S^{2}}\right)$$

(2) $$\text{KMO}=\frac{\sum \sum_{i\neq j}r_{i\,j}^{2}}{\sum \sum_{i\neq j}r_{i\,j}^{2}+\sum \sum_{i\neq j}r_{i\,j,1,2\,\ldots }^{2}}$$

(3) $$r_{i\,j\,\left(k\right)}=\frac{r_{i\,j}-r_{i\,k}\,r_{j\,k}}{\sqrt{1-r_{i\,k}^{2}}\,\sqrt{1-r_{j\,k}^{2}}}$$

(4) $$t=\frac{r\,\sqrt{n-q-2}}{1-r^{2}}$$

In Eqs (1–4), α is the reliability coefficient, *K* is the number of questions, $S_{i}^{2}$ is the score variation on question *i*, *S*^2^ is the variance of the total score, *r*_*ij*_ is the partial correlation coefficient between variables *i*,*j*, *r*_*jk*_ is the partial correlation coefficient between variables *i*,*k*, *n* is the partial correlation coefficient between variables *j*,*k*, *n* is the number of experimental samples and *q* is the order.

The value of Cronbach’s alpha coefficient based on the reliability test in this questionnaire is 0.96, and the half correlation coefficient is 0.85 ∼ 0.89, the KMO value reaches 0.92, and the reliability and validity of the questionnaire are good.

## Results

### Questionnaire Results

Table 6 shows the gender and age, and Table 7 shows the educational background and occupation of the respondents.

**TABLE 6:** Basic information of the respondents.

	Gender		Age			
	Male	Female	Under 22	22–25	25–28	Over 28
Number	96	72	64	45	35	24
Proportion of valid questionnaires	57.14%	42.86%	38.10%	26.79%	20.83%	14.28%

**TABLE 7:** Educational backgrounds and occupations of the respondents.

	Education				Occupation	
	Undergraduates	Post-graduates	Doctors	Others	Students	Teachers
Number	97	43	12	16	118	50
Proportion of valid questionnaires	57.74%	25.60%	7.14%	9.61%	70.15%	29.85%

This shows that the validity of the questionnaire is also good. Table 5 shows that the number of males is 24 more than that of females. The number of undergraduates is more than that of other groups because many questionnaires are sent to young people under 25. The proportion of post-graduates and doctors is about 30%. This proves that this survey is designed for IPE of college students.

### Influencing Factors of Ideological and Political Education

**FIGURE 5:**
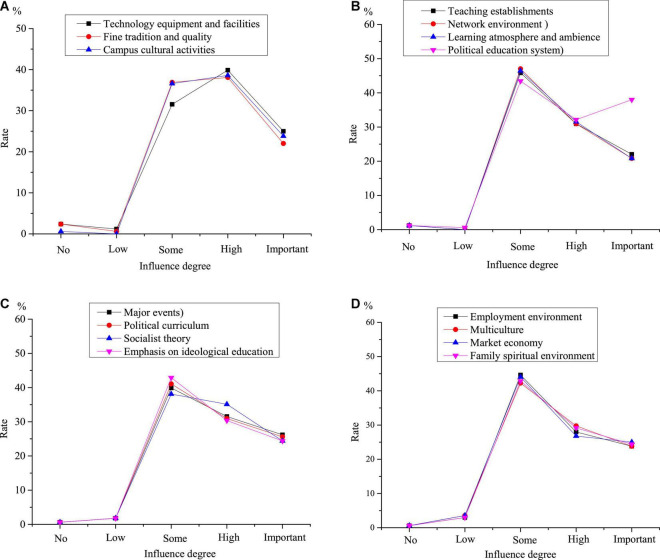
Influencing factors (A) positive factors (B) negative factors (C) opportunities (D) challenges.

Table 8 shows the teaching strategies of college students’ IPE based on the SWOT analysis, and Figure 5 shows the influencing factors of the teaching strategies of college students’ IPE.

**TABLE 8:** The SWOT analysis.

**Strengths** a. Technical equipment and facilities b. Excellent traditions and qualities c. Campus cultural life	**Weaknesses** a. Lack of teaching places for IPE b. Uncontrollable network environments c. Dull classroom atmosphere of IPE d. The imperfection of the assessment system of IPE
**Opportunities** a. Major events b. Curriculum reform c. New development of socialism theory with Chinese characteristics d. Much attention is given to IPE	**Threats** a. Severe employment environments b. Multiculturalism c. Negative impact of market economy d. Weakening family spiritual environments

Figure 5 shows that the positive factors have a great impact on implementing the teaching strategies of IPE. More than 97% of the respondents think that the positive factors have an impact on implementing the teaching strategies. Among them, more than 51% think they have a great impact, and more than 20% think they are the decisive factors. In terms of the negative factors, more than 98% of the respondents think they have an influence on implementing the teaching strategies, of which more than 52% think they have a great influence, and more than 22% think they are the decisive factors. 97% of the respondents believe that opportunities have an influence on implementing the teaching strategies, of which more than 51% think opportunities have a great influence, and 21% think they are the decisive factors. More than 97% think challenges have an impact on implementing the teaching strategies, of which more than 55% think they have a significant impact, and more than 24% think they are decisive.

### Discussion and Suggestions

The Party Central Committee and the State Council attach great importance to the work of IPE in colleges in the new era, and should give full play to the value’s guidance and role of IPE. Therefore, it is particularly important to improve the effectiveness of IPE in colleges. According to the relevant theories of value identity, combined with the structural and psychological mechanism characteristics of college IPE value identity revealed in the previous empirical research, it is essential to start from the following three aspects to effectively improve the effectiveness of college IPE.

#### Strengthen the Identity of the Identity Subject

Ideological and political education content should pay attention to the guidance of values and the cultivation of the sense of mission. When teaching the road, theory and system of socialism with Chinese characteristics, college IPE teachers should focus on grasping the pulse of the times and combine it with the needs of social development and hot social issues. In this way, college students can deeply understand the rich connotation of socialism with Chinese characteristics. In the specific teaching of the socialist system with Chinese characteristics, teachers should not stay at the macro level and excessively publicize the advantages of the socialist system. They should also let college students recognize the challenges faced in the road of socialist construction, let them dialectically analyze these problems, consciously and actively participate in the solution of practical problems, and enhance their sense of mission in participating in the construction of socialism with Chinese characteristics. Besides, college teachers of theoretical courses should integrate the national spirit and the spirit of the times into IPE courses. Since China’s reform and opening up, the spirit of the times with rich connotation has been formed with the efforts of countless socialist builders. Teachers should take specific cases as the starting point, teach the spirit of the times, and let college students continue to remember their sense of responsibility and mission as the successor of socialist builders under the guidance of these predecessors. In the process of long-term historical development, China has formed a national spirit with patriotism as the core. IPE values should integrate the great national spirit of the Chinese nation, so that college students can arouse a sense of pride and achievement as a Chinese from the bottom of their heart, consciously take their family and country as their own responsibility and shoulder the important task of the times.

#### Pay Attention to the Personal Needs of the Identity Subject

Ideological and political education should not be placed in a high position, but should move toward life and promote its value recognition. The needs of college students show the characteristics of multi-level and diversified development. IPE workers in colleges should grasp the common needs of college students and integrate IPE into them. The needs of college students in school are reflected in learning questions, life problems, and interpersonal relationships. Teachers should be good at actively integrating IPE methods and values education in the process of helping college students solve practical problems. For example, college students have poor pressure resistance and may lose confidence in the face of setbacks. IPE workers carry out necessary counseling after understanding the situation. Besides, they should integrate the thought of Marxist dialectics, so that college students can learn to dialectically analyze the two sides of setbacks, realize that the road is tortuous and the future is bright, and consciously establish firm ideals and beliefs.

#### Optimize the Psychological Path of Value Identification

Based on the structure of IPE value identity and the dimension characteristics of its psychological mechanism, IPE should follow the psychological mechanism of college students’ value identity: from cognitive identity to emotional identity, and then to internalized identity to behavioral identity. IPE should follow the psychological structure of college students’ value identity. In the process of cognitive identity, teachers should first clarify the meaning and content of IPE, eliminate the wrong cognition of college students, and let them have a clear and accurate positioning for IPE. Through the teaching of theory courses in colleges and the implicit education of college IPE workers, college students can accept ideological and political views. It is found that college students believe that IPE is useless. Given this phenomenon, it is considered that IPE should combine IPE content with life on the basis of scientization, strive to solve the practical problems of college students, enhance college students’ perceptual cognition of IPE, make college students change from passive acceptance to active acceptance, and establish the guiding significance of IPE for college students’ life. In the aspect of emotional identity, teachers should mobilize college students’ interests and hobbies in IPE. They should enhance college students’ emotional experience of IPE activities and make IPE move from rigid classroom mode to real colorful activities. In the stage of internalized identity, a link between IPE and college students should be established by combining projection and internalized identity theory.

## Conclusion

From the perspective of the structure and psychological mechanism of IPE value identity in colleges, the countermeasures to enhance IPE effectiveness are put forward. The following conclusions are drawn through investigation and analysis. The structure of IPE value identity in colleges is generally good, but there are different characteristics in four dimensions: cognitive identity, emotional identity, internalized identity and behavioral identity. Especially in terms of emotional identity, college students’ way recognition and content satisfaction with IPE are relatively low. Among demographic factors, political factors and school types significantly impact value identity. There are some problems in college IPE’s projection and transformation mechanism, such as college students’ vague cognition of IPE and the imperfect work of IPE workers in colleges. There is a lack of emotional resonance and the value conflict between IPE and college students in the internalization acceptance mechanism. In the mechanism of information externalization, college students mainly have the problem of separation of knowledge and behavior and weak willpower, resulting in a low degree of behavior recognition. The following measures are put forward in view of the above problems: optimizing the path of value identification and relying on emotional experience education methods.

Although the expected results are achieved, there are also some shortcomings: (1) only the college students in a university are investigated, which may affect the research conclusions; and (2) the survey is conducted without considering the influence of regions, which may also have an influence on the conclusion. In the follow-up study, the students in different colleges and universities will be investigated, and the influence of the regions will also be included.

## Data Availability Statement

The raw data supporting the conclusions of this article will be made available by the authors, without undue reservation.

## Ethics Statement

The studies involving human participants were reviewed and approved by the Ethics Committee of Northwestern Polytechnical University. The patients/participants provided their written informed consent to participate in this study. Written informed consent was obtained from the individual(s) for the publication of any potentially identifiable images or data included in this article.

## Author Contributions

The author confirms being the sole contributor of this work and has approved it for publication.

## Conflict of Interest

The author declares that the research was conducted in the absence of any commercial or financial relationships that could be construed as a potential conflict of interest.

## Publisher’s Note

All claims expressed in this article are solely those of the authors and do not necessarily represent those of their affiliated organizations, or those of the publisher, the editors and the reviewers. Any product that may be evaluated in this article, or claim that may be made by its manufacturer, is not guaranteed or endorsed by the publisher.
